# Effects of music on arousal during imagery in elite shooters: A pilot study

**DOI:** 10.1371/journal.pone.0175022

**Published:** 2017-04-17

**Authors:** Garry Kuan, Tony Morris, Peter Terry

**Affiliations:** 1 Exercise and Sports Science, School of Health Sciences, Universiti Sains Malaysia, Kubang Kerian, Kelantan, Malaysia; 2 College of Sport and Exercise Science, and Institute of Sport, Exercise and Active Living, Victoria University, Melbourne, Australia; 3 Division of Research & Innovation, University of Southern Queensland, Toowoomba, Australia; University of Marburg, GERMANY

## Abstract

Beneficial effects of music on several performance-related aspects of sport have been reported, but the processes involved are not well understood. The purpose of the present study was to investigate effects of relaxing and arousing classical music on physiological indicators and subjective perceptions of arousal during imagery of a sport task. First, appropriate music excerpts were selected. Then, 12 skilled shooters performed shooting imagery while listening to the three preselected music excerpts in randomized order. Participants’ galvanic skin response, peripheral temperature, and electromyography were monitored during music played concurrently with imagery. Subjective music ratings and physiological measures showed, as hypothesized, that unfamiliar relaxing music was the most relaxing and unfamiliar arousing music was the most arousing. Researchers should examine the impact of unfamiliar relaxing and arousing music played during imagery on subsequent performance in diverse sports. Practitioners can apply unfamiliar relaxing and arousing music with imagery to manipulate arousal level.

## Introduction

Music listening is associated with sport in several ways, including for entertainment, eliciting patriotism and pride, and enhancing the psychological state of athletes [1, 2]. The demonstrated benefits of music listening for athletes include arousal control, lowered perceived effort, improved affective states, synchronization effects, and enhanced performance [3, 4]. The present study focused on use of music for arousal control, extending the existing literature in the area. Burns et al. previously studied the effects of different types of music on relaxation levels, skin temperature, and heart rate when listening to classical, hard rock, self-selected relaxing music, and no music [5]. Classical and self-selected relaxing music increased subjective perceptions of relaxation more than hard rock music, but no differences were found on the physiological indicators of arousal, highlighting a disparity between perceived effects of music on arousal and objectively assessed effects. Our study investigated this disparity further.

Sports performance in training and competition can be highly arousing, with heart rate and mental alertness typically increasing significantly [6]. In some circumstances, such as during power, strength, or endurance tasks, this tends to have a positive effect on performance, whereas in activities requiring fine muscle control high arousal is typically detrimental to performance [7]. As arousal is so pervasive and an almost inevitable consequence of sport performance, understanding the arousal-performance relationship is of great interest to sport psychology practitioners seeking to help athletes gain the benefits of optimal arousal for peak performance.

Arousal can be measured centrally in the brain using an electroencephalogram, or peripherally autonomic measures, such as heart rate, peripheral temperature, blood volume pulse rates, and muscle tension, as indicators [8]. Arousal can be manifested in mental, physical, and behavioural reactions. In sports, over-arousal might cause behavioral changes leading to, for example, a highly-aroused soccer player showing decrements in passing performance due to poor decision-making, or incorrect skill execution caused by muscle tightness [8]. The present investigation explored the music-arousal link among a group of highly-skilled pistol shooters while they used imagery to mentally rehearse performance.

Imagery is a cognitive process in which individuals recall and create sensory experiences in the absence of external stimuli [9] and is among the most popular forms of mental training techniques used in psychological skills programs for athletes [10, 11]. Athletes often use imagery to prepare themselves for performance, mentally rehearsing routines related to their respective sports before a competition [12]. There is evidence to suggest that arousal-reducing music promotes more effective use of imagery [13] and many commercially-available CDs of guided imagery for peak performance [14, 15] incorporate imagery scripts with soft background music.

There is considerable literature on effective imagery use [11, 12, 16] but limited empirical evidence about using music to lower arousal during imagery. Dorney et al. examined effects of imagery with music and no music on a muscular endurance task and showed that imagery with music was associated with a significant increase in heart rate during preparation, but heart rate was unrelated to task performance [17]. Karageorghis et al. examined effects of voice-enhancing technology (VET) and relaxing music during imagery among break-dancers, and found that the VET plus relaxing music condition improved the efficacy of relaxation and motor performance compared to the control condition [18]. They concluded that use of imagery with music warranted further investigation in sport and exercise contexts.

To investigate the potential of relaxing and arousing music to enhance the impact of imagery on performance, it is first necessary to identify music that is consistently perceived to be relaxing or arousing. Thus, the purpose of the present study was to examine the effects of music on level of arousal, measured by physiological indicators *and* subjective ratings, while pistol shooters performed imagery of their shooting task.

### Stage 1: Identification of relaxing and arousing classical music excerpts

In Stage 1, preliminary procedures were completed to identify appropriate examples of relaxing and arousing music.

#### Music selection

All music used in this study can be termed classical music [19]. Although there is a broad variety of forms, styles, genres, and historical periods, classical music involves greater complexity than other types of music, such as folk, blues, and rock, and tends to be written and performed by those who understand notation and the written quality of music. Thus, although much classical music is highly arousing, with a driving beat that increases physiological and psychological states, the elements of music that stimulate arousal is derived from its structural notation. Classical music tends to have few changes in chord progression, unlike modern music, which often includes improvisation and rhythmic flexibility that are less suitable for imagery purposes [11]. Lowis found that listening to classical music, particularly fast or arousing pieces, can lead to reports of increased peak experiences [19]. According to Nercessian, relaxing classical music, characterized by a slower beat, with smoother and longer melodic patterns, decreases arousing emotions, creating a less tense, less formal, and less restrained environment for imagery, which generates physical relaxation and mental calmness that enhances cognitive responses and creativity. Nercessian proposed that arousing classical music is usually based on a powerful beat, and tends to have short melodic patterns that cause awakening and alertness, and stimulate energetic responses in people when they are using imagery [20].

In our study, we compared relaxing and arousing classical music that was judged to be unfamiliar to the general populations in order to minimize pre-existing associations, which can produce unpredictable effects on individual arousal levels. Although the focus of the present study was on unfamiliar classical music, researchers have suggested that familiar arousing classical music is particularly potent, because of its associations with arousing contexts [4]. Thus, we added this specific comparison to check whether familiar arousing music is significantly more powerful than unfamiliar arousing music. Given that we found no equivalent prior claim for familiar relaxing music in the literature, we considered this music condition to be unnecessary.

Music excerpts for arousing and relaxing conditions were selected using a systematic process. First, we selected 90 pieces of classical music recommended in the Australia Music Therapy Association lists, based on criteria determined in previous research [20, 21]. Three experienced music therapists who were also professional musicians with at least 10 years of orchestra experience, acted as expert judges. Second, we presented each judge with 30 classical musical excerpts in a quiet room, on three occasions, using a portable compact disc audio system with headphones, so that each judge considered all 90 pieces. The music was played at 55–70 decibels, which is within a pleasant hearing range for individuals with normal hearing [22]. After listening to each piece for one minute, the judges rated the music in terms of how relaxing or arousing they perceived it to be on a rating scale from 1 (very relaxing) to 10 (very arousing). Each visit lasted approximately one hour. Through this expert judging process, 72 excerpts were identified as appropriate for use as relaxing or arousing music during imagery, and 18 excerpts were rated as “less appropriate” due to potential confounding effects, including cultural interpretation, social influences, or excess variation in the melodic structure. Each judge independently organized the 72 music excerpts into categories representing “relaxing” and “arousing”, and “familiar” or “unfamiliar” music according to the perceptions of the general population. Four groups of music excerpts were produced, including 33 familiar relaxing excerpts, 17 unfamiliar relaxing excerpts, 12 familiar arousing excerpts, and 10 unfamiliar arousing excerpts. We excluded the familiar relaxing excerpts.

#### Psychological assessment of music

Three sport psychologists with at least two years experience of using imagery with athletes identified the three music excerpts they considered the general population would perceive to be unfamiliar relaxing music (URM), unfamiliar arousing music (UAM), and familiar arousing music (FAM). The nine music excerpts determined by consensus among the three sport psychologists (see Table 1) were pilot tested with five volunteer undergraduate students of sport sciences. All students confirmed they has not previously heard any of the six pieces of unfamiliar music. In terms of subjective experience, all students reported that the pieces intended to be arousing were actually arousing, and that the pieces intended to be relaxing were indeed relaxing. It was concluded that the nine selected pieces of music were appropriate for use in this study of the physiological and psychological effects of relaxing and arousing music during imagery among elite shooters.

**Table 1:** Preselected classical music excerpts.

Composer	Name
Unfamiliar Relaxing Music (URM):	
Copland	Appalachian Spring: Ballet for Martha
Delius	Florida Suite: III Sunset—Near the Plantation
Respighi	The Birds: The Dove
Unfamiliar Arousing Music (UAM):	
De Luca	Conquerors of the Ages: Attila the Hun
Mussorgsky	The Great Gate of Kiev
Shostakovich	Music from the Gadfly: Finale
Familiar Arousing Music (FAM):	
Orff	Carmina Burana: O Fortuna (orchestra only)
Tchaikovsky	1812 Overture: Grand Finale
Wagner	Die Walkure: The Ride of the Valkyries

### Stage 2: Effects of music on arousal during imagery among elite shooters

In Stage 2, we used a repeated measures design, in which each participant performed their usual imagery with URM, UAM, and FAM. During each session, we monitored physiological variables and after each session participants completed rating scales related to level of arousal, familiarity, and preference.

## Materials and methods

### Participants

Participants were 12 elite air pistol shooters (male = 8, female = 4), aged 22 to 41 years (M = 29.3, SD = 5.1), recruited from the Melbourne International Shooting Club and Victorian Amateur Pistol Association. All participants had at least two years of competitive experience at state level in air-pistol shooting. We chose shooting because it involves fine muscle movement that demands much lower levels of arousal during performance than high impact sports. To be eligible for inclusion, participants were required to have normal hearing and to score in the moderate-to-high imagery ability range on the Sport Imagery Ability Measure (SIAM) [23]. The required sample size was calculated using a G*Power 3.1 analysis. With a significance level of .05, a moderate effect size of .50 based on previous research [24], and statistical power of .80, 14 participants were required to provide adequate power [25]. However, due to the restricted number of eligible participants, 12 shooters were recruited.

### Measures

To measure the impact of the music excerpts on the arousal of skilled shooters, both physiological and psychological data were collected. Physiological signal data were collected using the ProComp+ system and BioGraph software version 5.0 from *Thought Technologies*^*TM*^ (Montreal, Canada), which assessed participants’ galvanic skin response (GSR), peripheral temperature (PT), and electromyography (EMG). EMG was collected at 256 Hz/secs, while GSR and PT were collected at 32 Hz/secs. Matlab software was used to combine the data into 10 second averages. Andreassi suggested that GSR, PT and EMG are good indicators of autonomic nervous system activity [26], providing researchers use non-invasive, reliable data sources to objectively evaluate physiological arousal level. To examine psychological perceptions, subjective ratings of arousal, music familiarity and music preference were assessed using a 100mm visual analogue scale from 0 (*relaxing*) to 100 (*arousing*), 0 (*not familiar*) to 100 (*very familiar*), and 0 (*dislike*) to 100 (*highly preferred*), respectively [27]. The pistol shooters also completed a short demographic form to collect information about gender, age, education, sports participation, and sports experiences.

#### Sport Imagery Ability Measure (SIAM)

The SIAM [23] assesses imagery ability in sport. Athletes imagine each of four generic sport scenes for 60 seconds each. The four scenes refer to the home venue, a successful competition, a slow start, and a training session. Following imagery of each scene, athletes respond to 12 items with reference to that particular scene. The 12 imagery ability items include five specific dimensions: control, vividness, ease, speed of generation, and duration, as well as six sensory modalities: kinesthetic, tactile, visual, auditory, olfactory, and gustatory senses associated with the image. In addition, one item assesses imagery of emotion experienced during each scene. Participants respond by placing a cross on a 100-mm analogue scale, anchored by opposing statements, such as “*no feeling”* and “*very clear feeling”* for the tactile modality of imagery. The 12 subscales appear in different orders for each scene to minimize order effects. The items comprising each dimension or modality are summed for each of the four scenes in the SIAM to create an overall score for each dimension or modality, which varies between 0 and 400 points. Watt et al. reported that the SIAM has been shown to have acceptable internal consistency, with alpha coefficients between 0.66 and 0.87 [23], and has been used frequently to screen for imagery ability in imagery studies [e.g., 7]. The SIAM was administered in the present study to screen participants for adequate imagery ability (at least moderate ability based on normative data in the SIAM Manual) to perform the shooting imagery tasks.

#### Music for imagery

The nine pieces of classical music were trimmed into three-minute excerpts and digitized into a laptop computer at Sound Work Music Recording Studio, Brunswick, Australia. Then, the music excerpts were prerecorded into a Sony Walkman digital media player (model ZWZZ1050B). A certified music audiologist from the studio evaluated both the Sony player and the headphones (Sennheiser’s HD 600 Avantgarde headphones) and fixed the sound at a moderate level and midrange on the volume dial, so the music did not cause discomfort or harm to the participants. This volume was chosen to ensure clear sound of the music, without raising it to a volume that would have been arousing.

#### Instructions for imagery

The following standardized instructions to participants were used: “Today you will be listening to three different types of music for three minutes; you will need to sit in silence for approximately 10 minutes, with electrodes placed on your non-dominant hand, and one electrode at your right frontalis muscle, with the headphone attached. To avoid inaccurate measurement, try not to move your non-dominant hand while performing the imagery. The music will then be played when your baseline of measures is achieved. When you hear the music sound in the background, you will slowly start to imagine your preparation routine during training, while listening to the music. As you get a picture of yourself performing normally the skill in practice, try to complete an entire training session successfully. Three minutes after the music finishes you will need to complete some questions. In between each session, you will have 10 minutes before the next music excerpt starts. Do you have any questions before we begin?”

### Procedure

The study was conducted in accordance with the Declaration of Helsinki and was approved by the Victoria University Human Research Ethics Committee. Participants were given an information sheet and provided written informed consent prior to data collection.

Music excerpts were randomly assigned to participants on three occasions prior to their normal training schedule in the testing room of the shooting club, which was quiet and unaffected by outside noise. Three pieces of music were played on each occasion. Participants were not informed about the pre-selection of the music as either relaxing or arousing. Prior to testing, participants avoided arousing activities, such as running, cycling, or swimming, to prevent the confounding potential of physical exertion on arousal. The temperature of the room was maintained in the range 20–24 degrees Celsius.

Participants sat in a comfortable chair while GSR, PT, and EMG sensors were attached and remained still and silent for up to 10 minutes while baseline readings of GSR, PT, and EMG (*t*_0_) were recorded. Arousal was monitored during imagery for each music condition (URM, UAM, FAM) in random order. Participants then recorded subjective ratings of arousal, music familiarity and music preference using the visual analogue scales. The total duration of each session was 60–80 minutes, depending on time taken for preparing participants, baseline readings, and for arousal level to return to baseline after each music excerpt.

### Data analysis

Data were compiled for analysis using SPSS 20.0 and checked for missing values, outliers, and non-normality. Means and standard deviations were calculated for each time point across the 12 shooters to examine trends for GSR, PT, and EMG from resting at 0 seconds (*t*_0_) to end of trial at 170 seconds (*t*_170_). We used readings at *t*_170_ as opposed to *t*_180_ (three minutes) because of the possibility of artefacts at *t*_180_ due to termination of the music. We identified the difference between the start and the end of each excerpt (*t*_170_) as the gain score (*t*_170—_*t*_0_). One-way Analysis of Variance (ANOVA) and post-hoc Tukey tests were used to assess differences among the three music conditions at the start of each excerpt (*t*_0_) and gain score for GSR, PT, and EMG. We also used ANOVA to compare ratings of subjective perceptions of arousal level, music familiarity and music preference across the different types of music.

## Results

### Imagery ability

Participants completed the SIAM as a measure of imagery ability. SIAM scores for the group were all above 260 points with a maximum of 400, except for the olfactory and gustatory subscales. These subscales appear to be less relevant to the present study, as smell and taste play a negligible role in shooting tasks. Scores above 200 are considered to reflect moderate levels of imagery ability, so scores indicated at least moderate imagery ability on all key subscales. This provided a clear indicator that all participants possessed adequate imagery ability to effectively employ imagery as part of their training program, so no participants were excluded from the study.

### Galvanic skin response (GSR)

**Fig 1:**
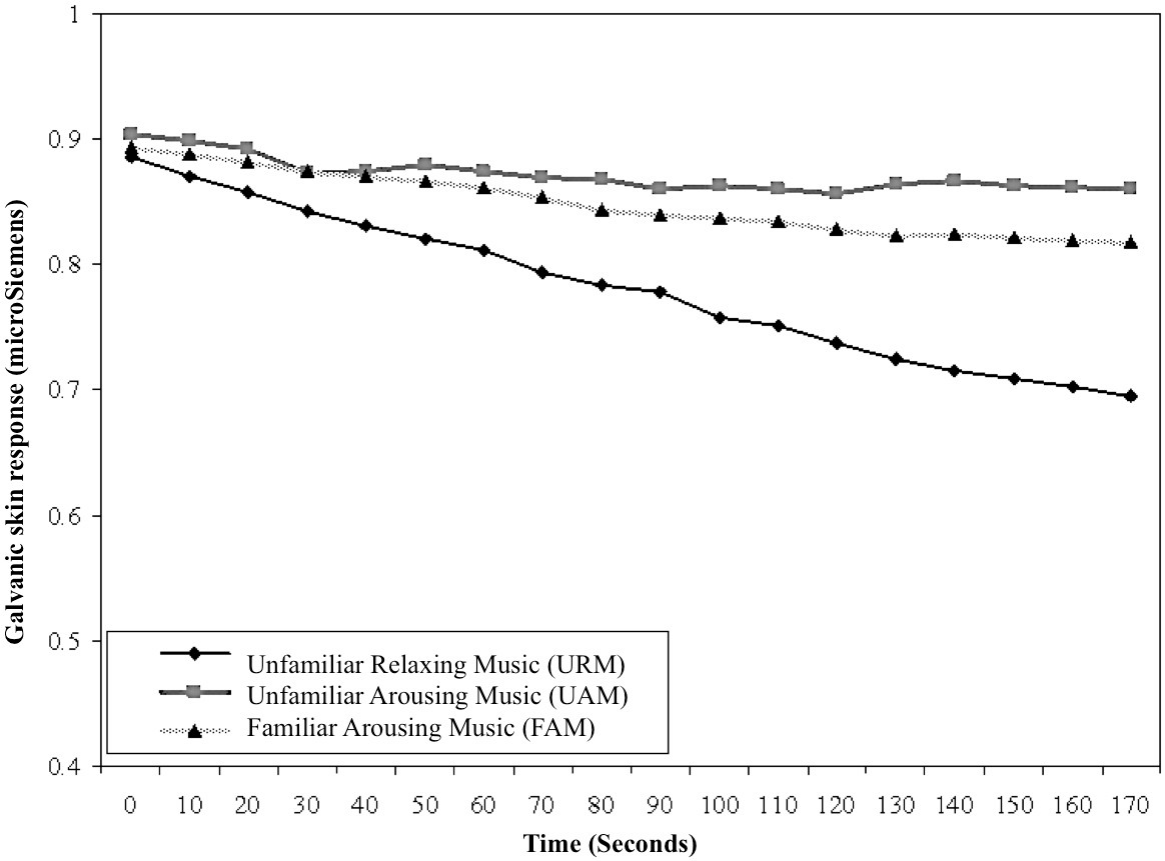
Galvanic skin response (GSR) for URM, UAM, and FAM. Peripheral temperature (PT).

The line-graph of GSR over time is presented in Fig 1 for URM, UAM, and FAM. All music conditions showed a decrease in GSR from *t*_0_ to *t*_170_ during imagery. URM showed the largest decrease in GSR level, whereas UAM and FAM showed lesser declines. ANOVA showed no significant baseline differences among the three music conditions, *F* (2,107) = -0.03, *p* = 0.97, eta^2^ < 0.001, whereas significant differences in gain scores of moderate magnitude were found among music conditions, *F* (2,107) = 9.26, *p* < 0.001, eta^2^ = 0.15. Tukey tests confirmed that URM (*M* = -0.19, *SD* = 0.16) was associated with significantly greater gain scores than either UAM (*M* = -0.04, *SD* = 0.14, *p* < .001) or FAM (*M* = -0.07, *SD* = 0.14, *p* = .005). There was no significant difference in gain scores between UAM and FAM (*p* = .66). Unfamiliar relaxing music was associated with a significantly greater decrease in GSR compared to the familiar and unfamiliar arousing music, indicating that it was more relaxing than either type of arousing music across the 3-minute excerpts. The absence of a significant difference in gain scores between UAM and FAM shows that the familiarity of the music did not influence changes in arousal level.

**Fig 2:**
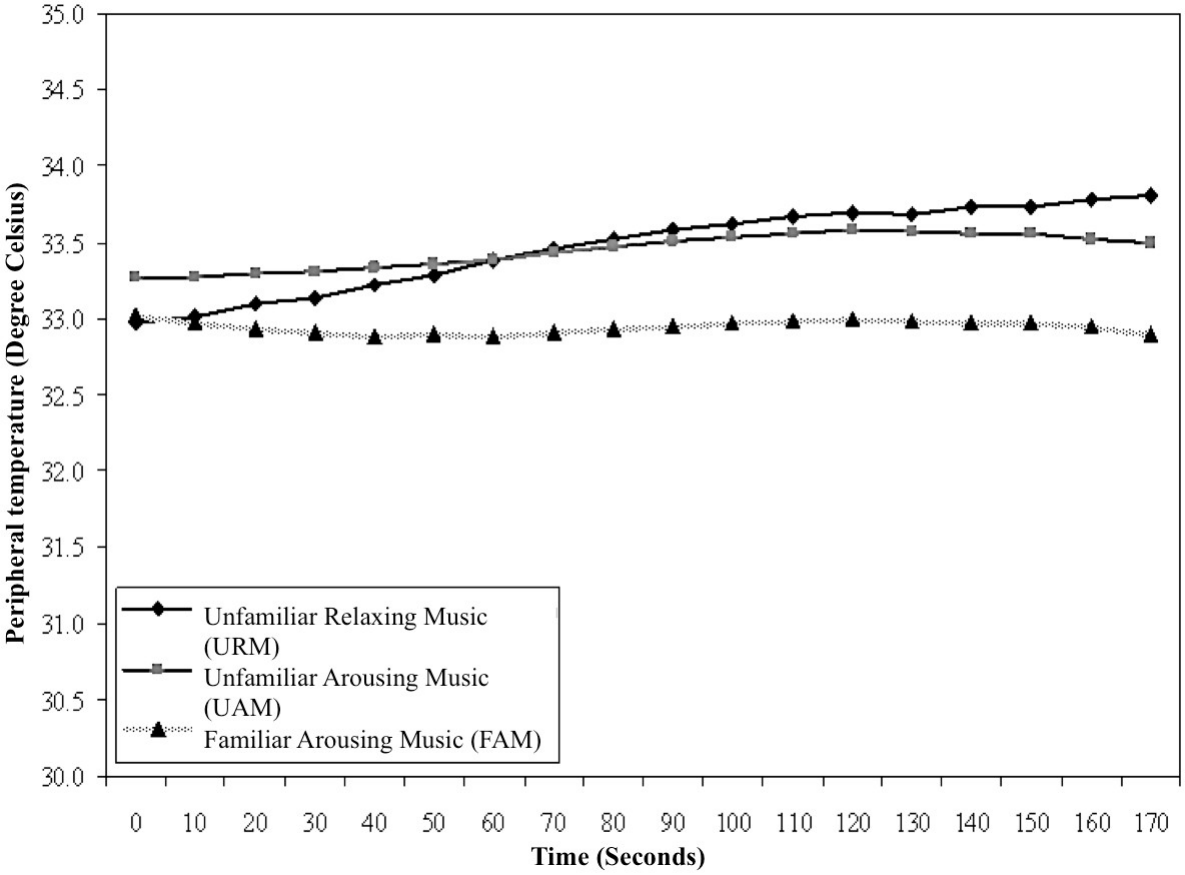
Peripheral temperatures (PT) for URM, UAM, and FAM. Electromyogram (EMG).

The line graph of PT over time is presented in Fig 2 for URM, UAM, and FAM. URM and UAM showed an upward trend in PT whereas the trend for FAM showed a slight decline. ANOVA showed no significant baseline differences among the three music conditions, *F*(2,107) = 0.52, *p* = .60, eta^2^ = 0.01, whereas a significant difference in gain scores of moderate magnitude was found between music conditions, *F*(2, 107) = 7.39, *p* = .001, eta^2^ = 0.12. URM was more relaxing (highest in PT level) than the two arousing music conditions. Tukey tests showed a significant difference in gain scores between URM (*M* = 0.83, *SD* = 1.21) and FAM (*M* = -0.11, *SD* = 0.86, *p* = .001) although no significant differences in gain scores were found between URM and UAM (*M* = 0.38, *SD* = 1.01, *p* = .18) nor between UAM and FAM (*p* = .11).

**Fig 3:**
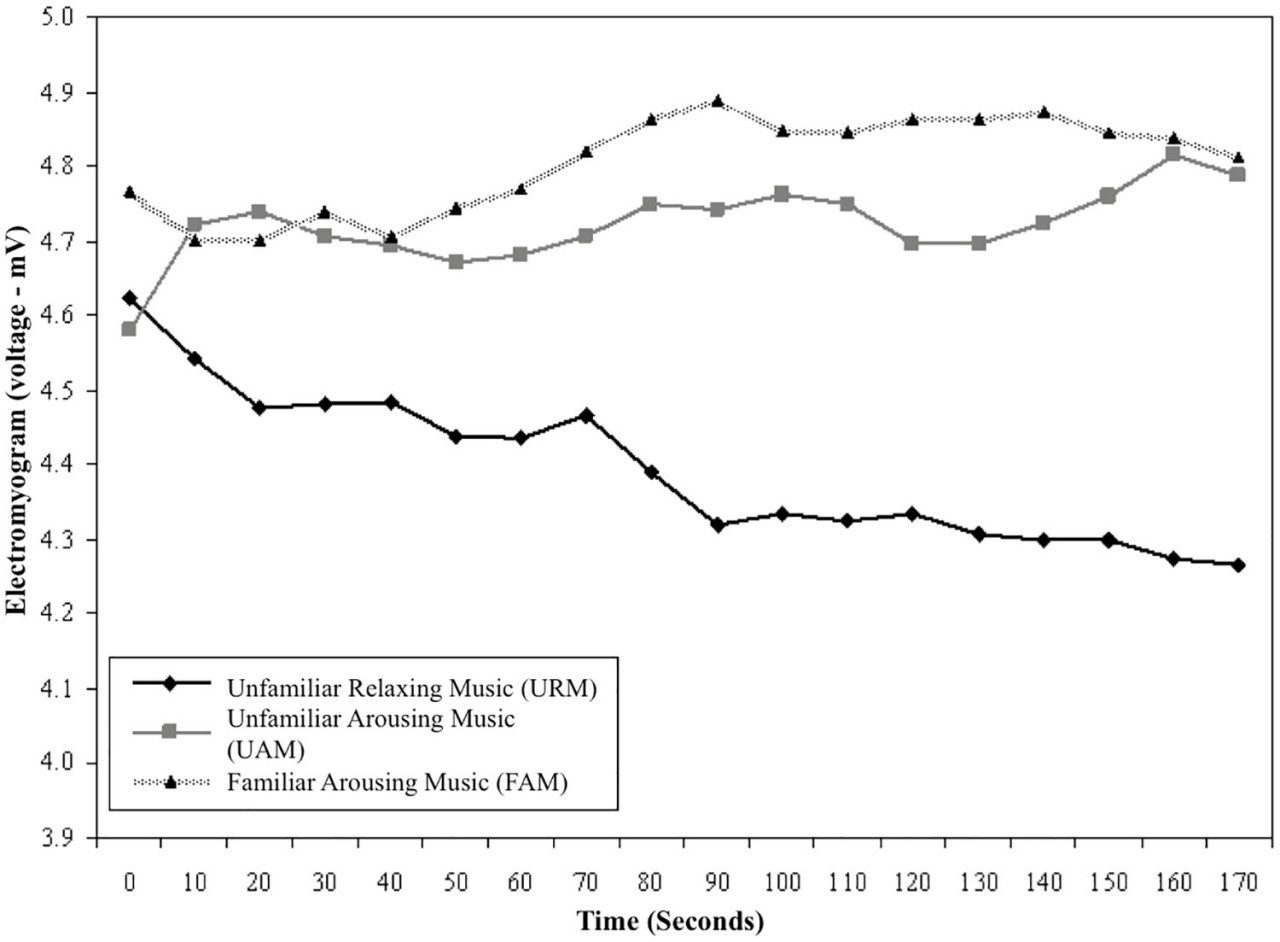
Electromyogram (EMG) for URM, UAM and FAM. Subjective perceptions of relaxation, familiarity, and preference.

The line-graph of EMG over time is presented in Fig 3 for URM, UAM, and FAM. URM showed a clear downward trend in EMG level from *t*_0_ to *t*_170_, whereas UAM and FAM showed slight increases. ANOVA showed no significant baseline differences among the three music conditions, *F*(2,107) = 1.02, *p* = .37, eta^2^ = 0.02, whereas a significant difference in gain scores of moderate magnitude was found between music conditions, *F*(2,107) = 9.21, *p* < .001, eta^2^ = 0.15. Tukey tests indicated significant differences between URM (*M* = -0.35, *SD* = 0.71), UAM (*M* = 0.22, *SD* = 0.56, *p* < .001), and FAM (*M* = 0.10, *SD* = 0.52, *p =* .005). Gain scores for UAM and FAM did not differ significantly (*p* = .69). These trends clearly show that URM was associated with a reduction in arousal level over the 3-minute period, whereas both UAM and FAM showed slight increases in arousal level.

Subjective ratings of arousal level, music familiarity, and music preference for URM, UAM, and FAM, are summarized in Table 2. UAM and FAM were rated toward the high end of the arousal rating scale, whereas URM was rated toward the low end of the scale. FAM was rated toward the high end of the familiarity scale, whereas URM and UAM were rated toward the low end of the scale. URM was given a high preference rating by participants, whereas both UAM and FAM were given moderate ratings.

**Table 2:** Means and standard deviations of ratings by music.

Variables	URM *M (SD)*	UAM *M (SD)*	FAM *M (SD)*	*F* (2,105)	*p value*
Arousal ratings	19.92 (13.01)	73.94 (13.56)	72.72 (13.73)	189.68	<.001
Familiarity ratings	24.52 (19.47)	24.26 (18.82)	74.33 (21.14)	76.17	<.001
Preference ratings	71.33 (15.17)	59.56 (21.11)	47.64 (27.24)	10.69	<.001

## Discussion

We examined the effects of classical music excerpts on physiological and subjective arousal indicators among 12 highly-skilled pistol shooters while performing imagery of their pistol shooting task. Results confirmed that the music excerpts selected had the hypothesized effects on arousal, based on continuous monitoring of physiological indicators and subjective ratings elicited after each excerpt. Using subjective analogue rating scales, participants reported that the music excerpts matched the proposed characteristics of familiarity and preference.

Findings confirmed the prediction that skilled pistol shooters would perceive URM to be more relaxing than either UAM or FAM. Trends for GSR, PT, and EMG across the 3-minute excerpts, averaged within type of music, showed greater reduction in arousal level for URM than for UAM or FAM. Correspondence between subjective assessments of reduced arousal and objective reductions in physiological indicators of arousal extended the work of Burns et al., who showed Mozart’s “Serenata Notturna” KV239 to be perceived as more relaxing than the hard rock track “So Close” by Alice in Chains, but found no differences in the physiological responses of participants [5]. The present investigation also extends the salient literature by virtue of having assessed physiological and psychological responses of relaxing and arousing music using solely classical music, most of which was unfamiliar to participants, and further by exploring both physiological and psychological responses to music of sports performers while using imagery.

All physiological measures showed relatively consistent patterns of changes to the shooters’ arousal during imagery sessions. Results were consistent with previous findings showing that listening to relaxing music during a fine-motor skill decreases arousal [28]. Results also supported the findings of Miluk-Kolasa and Matejek, who showed that listening to relaxing classical music helped return pre-surgical patients to a less physiologically-aroused state after learning about the surgical procedure they were to experience [29]. We observed an interesting pattern in GSR and EMG responses, which showed small decreases in arousal during UAM and FAM, rather than the anticipated increase in arousal typically found when such music is played prior to or during power and strength tasks [4, 30]. This indicated that arousal levels could decline, even when arousing music is played during imagery. One explanation for this could be that the task of imagery itself is associated with an automatic relaxation effect. If this is the case, athletes performing imagery may automatically relax when they become absorbed in their internal thoughts, which might counteract the influence of arousing music. Further research on this issue would be informative.

Results also showed that participants subjectively rated their arousal to be higher for UAM than for FAM and URM. The URM was least arousing, as predicted. Although FAM included famous arousing classical excerpts, they were not perceived to be more arousing than the UAM classical excerpts. We found no research in the literature comparing subjective responses to UAM and FAM classical music, either before or during a sports task or while athletes performed imagery, so this result should be replicated. Nonetheless present indications are that research examining relaxing and arousing music can be conducted with unfamiliar classical music, thereby minimizing the impact of past associations on individuals’ responses.

As predicted, participants rated familiarity for FAM to be higher than either URM or UAM. This confirms findings from the preliminary stage of the study in which musicologists and sport psychologists classified excerpts as either familiar or unfamiliar. Thus, participants confirmed that the familiar classical music was indeed more familiar among elite pistol shooters than the URM or UAM excerpts. Interestingly, participants rated URM as their highest preference when performing imagery, followed by UAM and FAM, in that order. This preference is consistent with the proposition that imagery is facilitated by relaxation [31].

We found GSR, PT, and EMG to be reliable physiological measures of level of arousal among fine-motor skill athletes. These physiological measures can be used in research to develop a clear and comprehensive understanding of the use of music with imagery for performance enhancement. However, the effectiveness of these physiological measures in detecting arousal effects of music during imagery of power, strength, or endurance tasks, which are inevitably arousing when athletes perform them, is not yet established.

### Limitations and future research

The use of shooters in the present investigation, who are typically located at the very low end of the arousal continuum during performance, may limit the generalizability of the findings. Pistol shooting involves minimal physical movement during performance which reduces the potential confounding effect of movement on assessments of arousal during imagery. Thus shooting can be viewed as an ideal sport for the present study, although whether the observed effects generalize to sporting tasks performed at higher levels of arousal remains uncertain, highlighting a need for replication in other sports.

We did not examine nor attempt to control the quality or content of imagery. All participants were skilled performers who habitually used imagery of pistol shooting in their normal training and competition routines. The focus of this study was to examine whether the music excerpts, especially the URM and UAM excerpts, had the predicted impact on physiological and psychological indicators of arousal during imagery. The shooters confirmed informally that they had used their usual imagery of performance during all nine excerpts of music. By using a repeated measures design, each participant acted as his/her own control. Thus, it is unlikely that the content of imagery changed in any systematic way that affected the results. Nonetheless, in further research, where performance of the sports task is a key outcome variable, imagery content should be controlled, so that variations in performance can be ascribed to the music conditions.

We used three physiological measures in this study—GSR, PT, and EMG—primarily because the *ProComp+* equipment is portable, measures all three indicators, and has been shown to be reliable for monitoring arousal level [5, 32]. This enabled us to test participants in a quiet room at the shooting club, which minimized travel time and ensured that they were not distracted by an unfamiliar environment. In addition, using several physiological indicators allowed for comparisons of patterns between measures to check that data were reliable. Although GSR, PT, and EMG all showed significant differences between music conditions, EMG showed greater variation than GSR and PT. EMG may be a less sensitive measure of general arousal than GSR or PT, because it is more readily affected by confounding variables, such as small movements. Furthermore, we were advised by shooting coaches to forego EMG measures in future studies, because of potential discomfort to participants.

We compared excerpts of three types of music played during imagery among elite pistol shooters. This is the first study that compared unfamiliar relaxing classical excerpts with unfamiliar arousing classical excerpts. Familiar arousing classical excerpts were added to test whether FAM was more arousing than UAM because of associations with the familiar excerpts. In extensive literature searches, we observed that most researchers who have studied music in sport used familiar music for their studies. In the present study, unfamiliar music was used to examine the arousing and relaxing properties of the music without the potentially confounding effects of familiarity and prior associations, or established preferences. Although results supported the use of unfamiliar relaxing and arousing music to achieve the aims of the study, future investigations using this kind of systematic approach are encouraged to further clarify the effects of familiar and unfamiliar music on arousal levels in general and specifically during imagery.

## Conclusions

GSR, PT, and EMG were effective as peripheral indicators of arousal although EMG was the least effective of these measures. The unfamiliar arousing and relaxing classical music pieces used in the present study were shown to be suitable for investigating effects of music on arousal during imagery.

## Supporting information

S1 Table Music_PlosOne. (XLSX)

S2 Table VAS_PlosOne. (XLSX)
